# The Interplay between Autophagy and Mitochondria in Cancer

**DOI:** 10.3390/ijms25179143

**Published:** 2024-08-23

**Authors:** Aleksandra Zdanowicz, Emilia Grosicka-Maciąg

**Affiliations:** 1 Department of Biochemistry, Medical University of Warsaw, Banacha 1 Str., 02-097 Warsaw, Poland; aleksandra.zdanowicz@wum.edu.pl; 2 Doctoral School, Medical University of Warsaw, Zwirki i Wigury 81 Str., 02-091 Warsaw, Poland; 3 Department of Biochemistry and Laboratory Diagnostic, Collegium Medicum Cardinal Stefan Wyszyński University, Kazimierza Wóycickiego 1 Str., 01-938 Warsaw, Poland

**Keywords:** mitochondria, mitophagy, autophagy, oxidative stress, cancer, autophagy-related biomarkers

## Abstract

Besides producing cellular energy, mitochondria are crucial in controlling oxidative stress and modulating cellular metabolism, particularly under stressful conditions. A key aspect of this regulatory role involves the recycling process of autophagy, which helps to sustain energy homeostasis. Autophagy, a lysosome-dependent degradation pathway, plays a fundamental role in maintaining cellular homeostasis by degrading damaged organelles and misfolded proteins. In the context of tumor formation, autophagy significantly influences cancer metabolism and chemotherapy resistance, contributing to both tumor suppression and surveillance. This review focuses on the relationship between mitochondria and autophagy, specifically in the context of cancer progression. Investigating the interaction between autophagy and mitochondria reveals new possibilities for cancer treatments and may result in the development of more effective therapies targeting mitochondria, which could have significant implications for cancer treatment. Additionally, this review highlights the increasing understanding of autophagy’s role in tumor development, with a focus on modulating mitochondrial function and autophagy in both pre-clinical and clinical cancer research. It also explores the potential for developing more-targeted and personalized therapies by investigating autophagy-related biomarkers.

## 1. Introduction: The Function of Mitochondria

Mitochondria are often termed the “powerhouse” of the cell because of their ability to transform the energy contained in glucose or other organic molecules into adenosine triphosphate (ATP).

**Figure 1:**
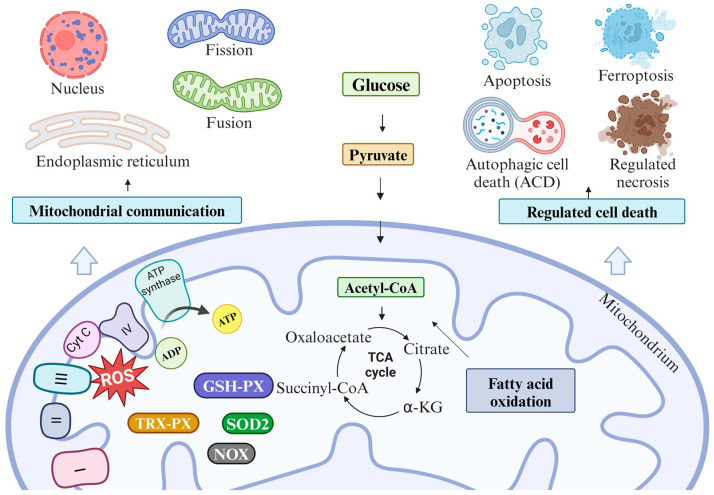
Overview of mitochondrial functions. Mitochondria perform several essential functions within the cell, including ATP production; reactive oxygen species (ROS) generation and elimination through superoxide dismutase (SOD), NADPH oxidase (NOX), catalase (CAT), glutathione peroxidase (GSH-Px), or thioredoxin peroxidase (TRX-Px); mitochondrial communication; and regulation of cell death. This image was created on BioRender.com.

During cellular respiration, apart from ATP production (Figure 1), mitochondria primarily generate reactive oxygen species (ROS), mainly superoxide (O^2−^), due to electron leakage at complexes I and III [1]. Under typical physiological conditions, mitochondria produce a modest level of mtROS (mitochondrial ROS), but in the presence of a mitochondrial dysfunction, the production of mtROS escalates uncontrollably. This excessive, unmanageable level of mtROS leads to modified mitochondrial redox signaling. Oxidative stress conditions within mitochondria are heralded by the impairment of the Krebs cycle, the degradation of mitochondrial proteins through their unfolding, also impacting cell death, mitochondrial DNA (mtDNA) mutations, and lipid damage [2]. Moreover, mitochondrial oxidative stress induces the expression of genes that activate stress response pathways, including the activation of Nrf2 (nuclear factor erythroid 2-related factor 2). The Nrf2 transcription factor controls the expression of antioxidant response genes related to glutathione, thioredoxin, iron metabolism, and NADPH production. Disruption of mtDNA by mtROS can impair ETC function because mtDNA encodes 13 mRNAs for mitochondrial respiratory complexes, 22 tRNAs, and 2 rRNAs [3]. The control of the accumulation and elimination of mtROS can be executed by either superoxide dismutase (SOD), NADPH oxidase (NOX), catalase (CAT), glutathione peroxidase (GSH-Px), or thioredoxin peroxidase (TRX-Px) [4]. The effectiveness of GSH-Px and TRX-Px is contingent upon the presence of reduced glutathione (GSH) and reduced thioredoxin (TRX). The replenishment of GSH and TRX relies on the functionality of reductases and the quantity of their cofactor, NADPH. Additionally, mtROS can be eliminated through the controlled removal of mitochondria, known as mitophagy [2].

Mitochondria play crucial roles in maintaining calcium homeostasis, the reprogramming of metabolism to suit physiological needs, and the regulation of the cell and organelles’ death. To maintain cellular homeostasis, mitochondria engage in communication with each other and with other organelles through various mechanisms, such as retrograde signaling, vesicular transport, signaling molecules, or direct interaction with the mitochondria-associated membrane (MAM) of the endoplasmic reticulum (ER). Collaboration with ER supports mitochondria in regulating Ca^2+^ transport, apoptosis, and phospholipids delivery. Additionally, cooperation with peroxisomes assists in fatty acid oxidation and the elimination of mtROS [5]. The formation of a mitochondrial network occurs through the processes of the fission and fusion of both the outer (OMM) and the inner (IMM) mitochondrial membranes. A proper balance between mitochondrial fusion and fission is vital for ensuring effective cell metabolism and adaptation to stress. The fusion of mitochondria facilitates the mixing of mtDNA and enhances MMP, mitochondrial respiration, and ATP production. Conversely, mitochondrial fission regulates apoptosis and mitophagy [6]. Furthermore, intact mitochondria possessing mtDNA are capable of transferring between cells via a mechanism termed horizontal mitochondrial transfer (HMT). The primary purpose of HMT is to share functional mitochondria with cells exhibiting aberrant mitochondrial functions. Consequently, cells receiving mitochondria restore mitochondrial respiration [3].

The ability of mitochondria to dynamically modulate and adapt cellular functions in response to stressful conditions is considered a pivotal factor in cancer development. The key difference in cancer cells’ mitochondria compared to those in healthy cells lies in the switching between the Warburg effect and OXPHOS, along with altered ROS production, disrupted calcium regulation, and aberrant interactions within the mitochondrial network [7]. This switch allows cancer cells to generate energy and biosynthetic precursors, enabling their rapid proliferation even in oxygen-deprived conditions. Alongside the Warburg effect, cancer cell mitochondria may undergo modifications in fatty acid oxidation and glutamine metabolism [8]. In cancer, mitochondria exhibit notable heterogeneity and dysregulation across various cellular processes, such as apoptosis, regulated necrosis, ferroptosis, and autophagic cell death (ACD). These characteristics significantly contribute to increasing the therapeutic resistance of patients [9].

The inhibition of mitochondrial function in malignant cells represents a promising strategy for selectively targeting malignant cells and reducing their adaptation to the tumor microenvironment, thereby impeding tumor progression. Mitochondria-targeted cancer therapy relies on distinctions between mitochondrial function in cancerous and healthy cells. One widely employed therapeutic approach involves inhibiting ATP production by suppressing either complex I or II in ETC. This generates significant energy stress in cancer cells, resulting in the initiation of autophagic cell death. Additionally, mitochondrial respiration can be inhibited by targeting specific enzymes in the TCA cycle, such as α-ketoglutarate dehydrogenase (KGDH) or pyruvate dehydrogenase (PDH) [10]. Another approach to targeting mitochondria in cancer involves promoting oxidative stress or inducing autophagy.

## 2. Autophagy

Originally, autophagy was defined as a cell survival mechanism that occurs under starvation or oxidative stress (hypoxia, ROS) conditions, wherein the cell degrades damaged or unnecessary organelles to generate nutrients. Recently, it was discovered that autophagy is involved in the quality control of organelles/proteins by degrading dysfunctional proteins/organelles. Autophagy is divided into selective and nonselective types. Nonselective autophagy is a process that transforms cellular energy by randomly removing organelles. In contrast, selective autophagy specifically targets damaged organelles, such as the ER, mitochondria, and peroxisomes, as well as cellular proteins. Nonselective autophagy is triggered under starvation or nutrient deprivation conditions, while selective autophagy is more prevalent in nutrient-rich environments [11].

It was recently reported that autophagic cell death (ACD) is likely an outcome of either extensive autophagy, prolonged stress, or inhibited apoptosis [12]. During ACD, nuclear condensation increases, caspase activity decreases, and cellular vacuoles are generated [13].

**Figure 2:**
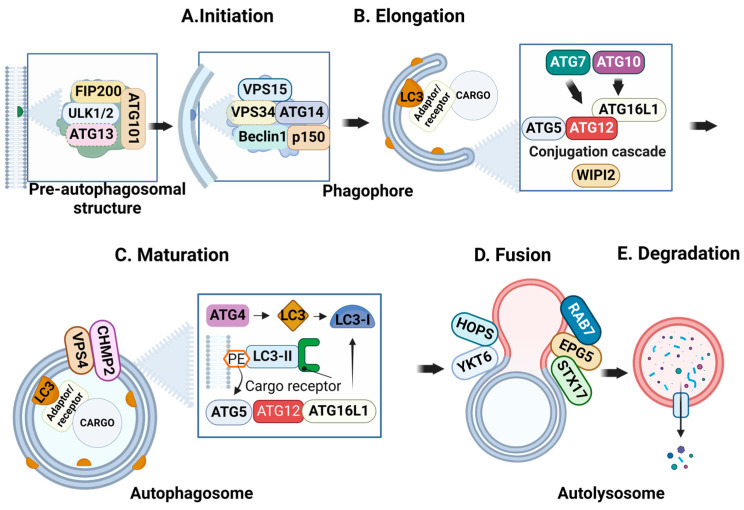
The autophagy pathway, which is subdivided into the following steps: (**A**) Initiation—the ULK complex, consisting of FIP200, ULK1/2, ATG13, and ATG101, mediates pre-autophagosomal structure formation. (**B**) Elongation—the PI3K complex, consisting of VPS15, VPS34, ATG14, Beclin1, and p150, generates the phagophore. Phagophore elongation is mediated by WIPI2, conjugation cascade ATG5-ATG12-ATG16L, ATG7, and ATG10. (**C**) Maturation—ATG4 convertss LC3 (an ATG8 family protein)-to-LC3-I and the conjugation cascade attaches LC3-I to PE (phosphatidylethanolamine) in the lipid membrane and generates lipidated LC3-II. The autophagosome’s closure is mediated by VPS4 and CHMP2 proteins. (**D**) Fusion—autophagososme–lysosome fusion is initiated by SNARE proteins (YKT6, STX17), tethering factors (HOPS, EPG5), and GTPase (RAB7). (**E**) Degradation. This image was created on BioRender.com.

Selective autophagy is a multistage process that includes the following steps: initiation, elongation, maturation, fusion, and degradation. Initiation (Figure 2A) commences with the activation of the ULK (Unc-51-like kinase) complex. This complex controls the commencement of selective autophagy by forming a stable protein assembly that includes ULK1/2 (serine/threonine Unc-51 like kinase 1/2), FIP200 (also known as RB1CC1), ATG13, and ATG101. Once this complex is formed, it initializes and regulates the formation of the pre-autophagosomal structure (PAS) and recruits ATG9 vesicles [14], which facilitate PAS expansion and are often referred to as the “seeds” of autophagosome formation [15]. The activity of the ULK complex is regulated by leading regulators of nutrient stress sensors, namely, mechanistic target of rapamycin complex 1 (mTORC1) and AMP-activated protein kinase (AMPK) [16]. Specifically, mTORC1 suppresses the function of the ULK complex through the phosphorylation of ATG13 and ULK1 proteins [17]. Furthermore, mTORC1 modulates the location of transcription factor EB (TFEB) by directing it to lysosomes. The key regulator of the autophagy pathway, TFEB, orchestrates the transcriptional regulation of autophagy genes via binding to the CLEAR (coordinated lysosomal expression and regulation) element in the nucleus. The interaction between TFEB and CLEAR promotes autophagy and facilitates lysosomal biogenesis [17]. The initiation of autophagy requires either the inactivation of mTORC1 or the activation of AMPK. The activity of AMPK depends on the availability of cellular energy. When ATP is depleted, AMPK phosphorylates ULK1 and inhibits the activity of mTORC1 [18].

The complex ULK initializes the formation of the second autophagic complex, also known as the PI3K (phosphatidylinositol 3-kinase) complex. It is composed of VPS34 (Vacuolar protein sorting 34, a class III Pi3K), VPS15, Beclin1 (Bcl-2-interacting myosin-like coiled-coil), ATG14, p150, and NRBF2 (nuclear-receptor-binding factor 2). This complex is engaged in the extension of the phagophore (an isolated lipid double-membrane structure) and the production of phosphoinositide 3-phosphate (PI3P) on the autophagic membrane (Figure 2). The elongation of the phagophore (Figure 2B) is initiated by the production of PI3P and the recruitment of the scaffold protein WIPI2 (WD-repeat domain phosphoinositide-interacting 2). Subsequently, WIPI2 facilitates the tethering of the endoplasmic reticulum membrane and phagophore via ATG2 and supports the assembly of the ATG5-ATG12-ATG16L1 complex, along with ATG3 and ATG7. These proteins are essential for the expansion and formation of the phagophore membrane, as they provide a physical platform and facilitate the lipidation of ATG8 family proteins (LC3 (microtubule-associated protein 1 light chain 3) or GABARAP (GABA type A receptor-associated protein)) [19]. The ubiquitin-like proteins LC3 and GABARAP facilitate the attachment of the cargo adaptor/receptor to the lipid membrane (specifically to phosphatidylethanolamine (PE)), acting as a binding platform [17]. The protein ATG4, along with its partners ATG7 (ubiquitin-activating enzyme (E1)) and ATG3 (ubiquitin conjugation enzyme (E2)), facilitate the transformation of LC3 into its soluble form, LC3-I [20]. The ATG5-ATG12-ATG16L conjugation cascade is essential for attaching soluble LC3-I to PE in the lipid membrane. This attachment leads to the generation of lipidated LC3-II, which acts as a docking site for autophagy cargo adaptors/receptors. But on the outer membrane, LC3-II promotes autophagosome maturation (Figure 2C) and lysosome merging. Autophagic cargo adaptors in the autophagosomal membrane, including p62/SQSTM1 and NRB1, selectively identify and associate with ubiquitinated cargo material [13].

The autophagosome membrane is closed by the activity of ESCRT components (endosomal sorting complex required for transport)—CHMP2 (charged multivesicular body protein 2) and VPS4 [21]. The Fusion (Figure 2D) of mature autophagosome with lysosomes is initiated by SNARE (soluble N-ethylmaleimide-sensitive attachment protein receptors) superfamily proteins, including STX17, SNAP29, VAMP3, VAMP7, VAMP8, and YKT6 [14]. They are both found on the autophagosome and lysosome membranes [22]. The process of fusion between the autophagosome and lysosome is promoted by tethering factors, including the HOPS (homotypic fusion and protein sorting) complex (VPS11, VPS18, VPS33A, VPS39, and VPS41), PLEKHM1 (pleckstrin-homology-domain-containing family M member 1), and EPG5 [23]. The tethering factors interact with GTPases, including ARL8B and RAB7, located in the lysosome membrane. Additionally, they bind to ATG8 family proteins present on the outer membrane of autophagosomes [22]. After the autophagosome fuses with the lysosome, the autophagic cargo undergoes degradation (Figure 2E) mediated by lysosomal enzymes. This process causes the degradation of organelles and the release of metabolic components.

### 2.1. Selective Autophagy—Mitophagy

Selective autophagy is a process that selectively, i.e., according to specific receptors, targets and degrades old and dysfunctional organelles, including mitochondria (mitophagy), ribosomes (ribophagy), peroxisomes (pexophagy), and the endoplasmic reticulum (ER) (reticulophagy).

The primary purpose of mitophagy is to regulate the quality and quantity of mitochondria via the selective elimination of dysfunctional or damaged organelles [24], and it is important for embryonic development, apoptosis, and cell differentiation. The main role of mitophagy is to maintain mitochondrial quality control and homeostasis. When mitophagy is ineffective at removing damaged mitochondria, the accumulation of dysfunctional or deficient mitochondria increases, resulting in decreased OXPHOS performance and increased levels of ROS. This imbalance can disrupt metabolism, cause cellular damage, and eventually result in cell death. Improper mitophagy contributes to the development of various pathological states, such as cancer and metabolic, neurodegenerative, cardiovascular, and skeletal muscle diseases [25]. Mitophagy can be activated by nutrient limitation or mitochondrial dysfunction, among which the latter is caused by different types of stress factors, such as mtDNA damage, elevated levels of mtROS, misfolded mitochondrial proteins, hypoxia, and declined mitochondrial membrane potential (MMP) [26]. Mitophagy is divided into ubiquitin-dependent (PARKIN-dependent and -independent) and ubiquitin-independent (receptor-based) types.

#### 2.1.1. Ubiquitin-Mediated Mitophagy

**Figure 3:**
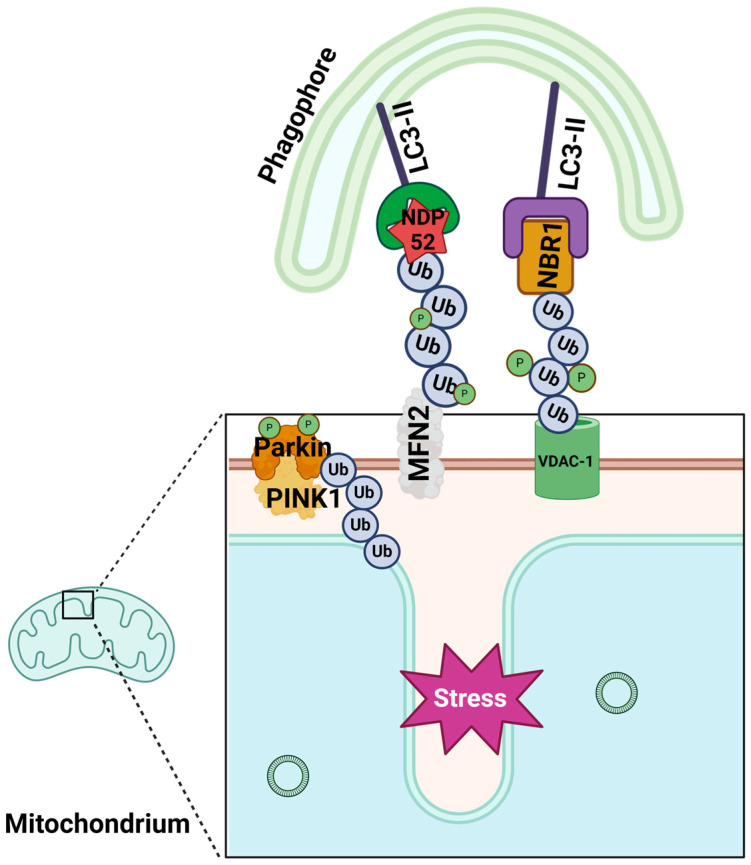
PINK1/Parkin-dependent mitophagy. Under conditions of mitochondrial stress, PINK1 accumulates on the outer mitochondrial membrane (OMM), where it undergoes phosphorylation and activation. This activation subsequently triggers Parkin ligase, which ubiquitinates mitochondrial proteins, such as MFN2 and VDAC-1. Mitochondria with polyubiquitinated OMM proteins recruit mitophagy cargo receptors/adaptors, such as NDP52 and NBR1, to their surfaces. The mitophagy cargo receptor/adaptors connect both with LC3-II, present on the phagophore membrane, and with ubiquitinated chains. This image was created on BioRender.com.

Ubiquitin-dependent mitophagy is a selective mechanism wherein mitochondria that are specifically marked with ubiquitin molecules are removed from the cell [26]. The initiation of PINK1 (PTEN-induced putative kinase 1)/Parkin-mediated mitophagy occurs in response to mitochondrial stress, including mitochondrial membrane depolarization or the extreme misfolding of mitochondrial proteins (Figure 3). Mitochondrial dysfunction leads to the accumulation of the serine/threonine PINK1 kinase at the OMM, whereas under physiological conditions, the levels of PINK1 in mitochondria are typically low. The durability of PINK1 protein at the OMM depends on the activity of TOMM7 (translocase of outer mitochondrial membrane 7), Hsp70 (heat shock protein), PHB2 (prohibitin 2), and PGAM5 (mitochondrial serine/threonine protein phosphatase) [27]. At a damaged OMM, PINK1 auto-activates, dimerizes, and induces cytosolic Parkin ligase (U3 ubiquitin ligase) activity via phosphorylation. The active Parkin ligase produces ubiquitin chains and tags OMM proteins, such as MFN2 (Mitofusin-2), VDAC-1 (voltage-dependent anion channel-1), and Miro (mitochondrial Rho GTPase), for mitophagy. Ubiquitinated OMM proteins provide a scaffold for the binding of autophagy adaptor proteins (also named autophagy receptor proteins). Besides Parkin ligase, other E3 ubiquitin ligases like GP78 (glycoprotein 78), MUL1 (mitochondrial E3 ubiquitin ligase 1), and SIAH1 (seven in absentia homolog 1) are also responsible for ubiquitinating mitochondrial proteins [24]. The tagged mitochondria are isolated from the healthy mitochondrial network through the activity of RHOT1 (Ras homolog family member T1) [28]. However, some studies have indicated that mitochondria are fragmented and separated from the ER before they can be tagged for mitophagy [29]. Additionally, mitochondrial proteins that are improperly tagged with ubiquitin chains are modified by deubiquitinase (DUB) enzymes [26].

In the next step, PINK1 phosphorylates ubiquitin chains that are attached to OMM proteins to enhance the mitophagy process. Finally, OMM proteins marked with ubiquitin are recognized by autophagy receptors/adaptors of ubiquitin-dependent mitophagy SQSTM1/p62 (sequestosome 1), OPTN (optineurin), CALCOCO2/NDP52 (calcium binding and coiled-coil domain 2), NBR1 (neighbor of BRCA1 gene 1 protein), AMBRA1, and TAX1BP1 (Tax1 binding protein 1) [25] (Table 1). At the N-terminal arm, these receptors possess an LC3-interacting region (LIR) that enables them to interact with the MAP1LC3/LC3 (microtubule-associated protein 1 light chain 3) and LC3 protein families, which are anchored in the phagophore membrane. The mitophagy receptors/adaptors can bind to the ubiquitinated OMM proteins through their ubiquitin-binding domain, thus forming mitophagy cargo. Once the mitophagic cargo binds to the mitophagy receptor, it triggers the formation of the mitophagosome. The mitophagosome, which encapsulates the marked mitochondrium, subsequently merges with lysosomes [30].

#### 2.1.2. Ubiquitin-Independent Mitophagy

Ubiquitin-independent mitophagy is a process where damaged mitochondria are directed to the mitophagosome without relying on ubiquitin tagging. Instead, this process depends on the presence of mitophagy receptors/adaptors, as summarized in Table 1. These receptors/adaptors directly interact with MAP1LC3/LC3 and LC3/GABARAP, which are localized on the phagophore membrane [31]. Ubiquitin-independent mitophagy receptors/adaptors, such as BNIP3 (BCL2/adenovirus E1B 19 kDa protein-interacting protein 3), BNIP3L/NIX, FUNDC1 (FUN14 Domain-Containing 1), BCL2L13 (BCL2-Like 13), FKBP8 (FKBP Prolyl Isomerase 8), and AMBRA1 (the activating molecule in BECLIN1-regulated autophagy 1), are directly located on the mitochondria membrane. The expression levels of these receptors are regulated by transcriptional and post-transcriptional modifications [26]. Besides protein mitophagic receptors, mitochondria also exploit lipid-based receptors like cardiolipin (CL) and ceramide [30] (Table 1). CL is typically found in the IMM, where it supports the activity of ETC complexes I, III, and IV and ATP synthase. However, under mitochondrial stress, CL can translocate to the OMM [32].

**Table 1:** Mitophagy receptors.

Type of Mitophagy Receptors	Key Properties	Regulators (Positive [+]/Negative [-])	Ubiquitin Dependent (+/-)	Location
Protein receptors				
SQSTM/p62	Oxidative-stress-inducible protein; regulator of Nrf2 factor, NF-kB [33]	ULK1 [+]; CK2 [+]; TBK1 [+]; mTORC1 [+];	+	Cytoplasm
CALCOCO2/NDP52	Promotor of phagophore biogenesis [34]	TANK binding kinase 1 (TBK1), [+];	+	Cytoplasm
OPTN (Optineurin)	Promotor of phagophore biogenesis [35]	TNF-α [+]; Interferons [+];	+	Cytoplasm
NBR1	Oxidative-stress-inducible protein; cooperates with p62 [35];	-	+	Cytoplasm
TAX1BP1	Eliminator of excessive ROS [36,37]	-	+	Cytoplasm
BNIP3	BCL2 apoptosis regulator protein (pro-apoptotic); promotes hypoxia-induced autophagy; regulator of mTOR [32];	FOXO3 (starvation, [+]); HIF-1 [+]; MA-5 [+];	-/+	OMM
BNIP3L/NIX	Stress sensor; inducer of cell death and mitophagy (ischemia, erythrocyte development [38])	HIF1A (hypoxia, [+]); GTPase RHEB (phosphorylation, [+]); PKA (phosphorylation, [-]);	-/+	OMM
FUNDC1	Involved in hypoxia-induced mitophagy; dephosphorylated form activates fission and mitophagy; connects with fission (DNM1L, DRP1)/fusion (OPA1) proteins; regulator of proteostasis [39]	SRC (kinase, [-]); CK2 (kinase, [-]); ULK1(kinase, [+]); PGAM5 (phosphatase, [+]); MARCH5 (ubiquitin ligase E3, [-]); MIR137 [-];	-	OMM
BCL2L13	Promotor of mitochondrial fragmentation; regulator of fission; maintainer of mitochondrial quality; inducer of apoptosis [32,40]	Unknown;	-	OMM
FKBP8	Anti-apoptotic protein; regulator of mTORC1; exerts peptidylprolyl isomerase activity [32,41]	RHEB [-];	-	OMM
AMBRA1	Phagophore activator; along with BECN1, it is an activator of PtdIns3k (phosphatidylinositol 3-kinase); interacts with HUWE1 (E3 ubiquitin ligase) and thus induces ubiquitin-independent mitophagy [32,42]	NFKBI [+]; BCL2 family proteins [-]; MCL1 [-]; CHUK [+]; GSK3B [+];	-/+	OMM
PHB2 (prohibitin 2)	Regulator of mitochondrial proteases; maintainer of mitochondrial genome; promotor of PINK1-PARKIN-dependent mitophagy; along with AURKA, it is a kinase promotor of PARKIN-independent mitophagy [32]	AURKA [+]	-/+	OMM/IMM
Lipid receptors				
CL (cardiolipin)	Maintainer of electron transport chain function; involved in apoptosis; cooperates with BECN1 (mitophagy) and DNM1L (mitochondrial division) [43]	CRLS1 (cardiolipin synthase 1, [+]); PLSCR3 ((phospholipid scramblase 3), transport CL to OMM [+]); NDPK-D (kinase, [+]) PRRT2/PKC [+]; SNCA[+];	-	OMM/IMM
Ceramide	Ceramide-induced mitophagy [32]	CERS1(ceramide synthase 1, [+]); DNM1L [+];	-	OMM

#### 2.1.3. Chaperone-Mediated Autophagy

**Figure 4:**
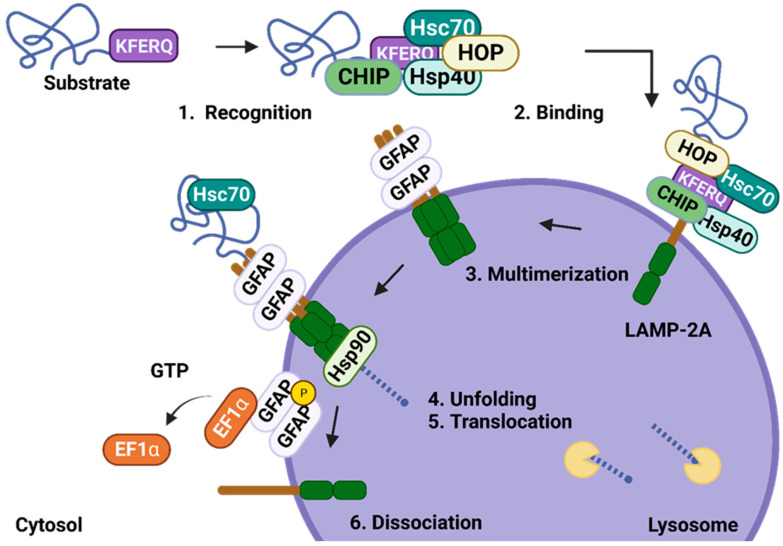
Mechanism of CMA (chaperone-mediated autophagy): 1. Recognition: Hsc70 and co-chaperons, such as Hsp40 (heat shock protein 40), CHIP (carboxyl terminus of hsc70-interacting protein), and HOP (hsp70-hsp90 organizing protein), recognize CMA substrates by specifically targeting the KFERQ motif present within the substrate. 2. Binding: CMA substrate binds to the lysomal surface receptor LAMP-2A (lysosome-associated membrane protein type 2A). 3. Multimerization: LAMP-2A forms a translocation channel through LAMP-2A multimerization and stabilization via GFAP (glial fibrillary acidic protein). 4. Unfolding: CMA substrate is unfolded by Hsc70 and stabilized by Hsc90. 5. Translocation: CMA substrate is transported into lysosomal lumen. 6. Dissociation: upon phosphorylation by EF1α, GFAP undergoes dissociation from the LAMP-2A channel, resulting in the disassembly of the LAMP-2A multimeric complex into monomeric form. This image was created on BioRender.com.

Chaperone-mediated autophagy (CMA) is a distinctive type of selective autophagy responsible for the lysosomal degradation of misfolded or damaged proteins. In contrast to ubiquitin-dependent/independent autophagy, during the CMA process, proteins are not enclosed in the autophagosome; instead, they are directly targeted by the chaperone Hsc70 (heat shock cognate protein 70) in the cytosol. The mechanism of CMA is categorized into distinct stages (Figure 4): 1. substrate recognition; 2. the binding of the substrate to the lysosomal membrane; 3. the multimerization of translocation complex; 4. substrate unfolding; 5. substrate translocation and degradation in the lysosomal lumen; and 6. dissociation of the translocation complex. Hsc70 recognizes CMA’s substrates by binding to the KFERQ motif in target proteins [44]. The function of Hsc70 is supported by various co-chaperons, including Hsp40 (heat shock protein 40), CHIP (carboxyl terminus of hsc70-interacting protein), BAG1 (Bcl2-associated athano-gene 1 protein), and HOP (hsp70-hsp90 organizing protein) [45]. In addition to recognizing substrates, the complex HOP is actively engaged in protein stabilization and unfolding [46]. After the substrate recognition step, Hsc70, along with the target protein, binds to the lysosomal receptor LAMP-2A (lysosome-associated membrane protein type 2A) [47]. LAMP-2A forms a dynamic translocation channel via multimerization and interaction with GFAP (glial fibrillary acidic protein), which is responsible for maintaining the structure of the multimeric LAMP-2A complex in the lysosomal membrane. Consequently, the CMA substrate is unfolded and transported into the lysosomal lumen, where it undergoes immediate degradation by lysosomal proteases [48]. After CMA substrate translocation, the interaction between GFAP and EF1α (elongation factor 1-α) is disrupted by GTP (guanosine triphosphate). This disturbance results in the release of EF1α to phosphorylated GFAP, which, in turn, facilitates the disassembly of LAMP-2A into its individual monomers [49]. The phosphorylation of GFAP on the lysosomal membrane supports the dissociation of GFAP from the LAMP-2A multimeric complex through the dimerization of phosphorylated GFAP with unmodified GFAP. The level of GFAP phosphorylation is controlled by the activity of AKT1 (AKT serine/threonine kinase 1), which becomes active when phosphorylated by mTORC2 (mammalian target of rapamycin complex 2) and inactive when dephosphorylated by PHLPP1 (PH domain and leucine-rich repeat protein phosphatase 1) [45].

The transport of LAMP-2A to the lysosomal surface represents a crucial stage in CMA. Moreover, the level of LAMP-2A directly affects CMA activity. The translocation of LAMP-2A to lysosomal membrane is determined by the involvement of Rab-7A (Ras-related protein Rab-7A), Rab-11A, DYNC1LI2 (Dynein Cytoplasmic 1 Light Intermediate Chain 2), and RILP (Rab-interacting lysosomal protein) [45]. CMA activity is regulated by the amounts of the key CMA proteins (Hsc70, GFAP, and LAMP-2A), as well as by the kinase AKT1 and circadian cycle regulators, such as BMAL1 (basic helix–loop–helix ARNT-like 1), PER1/2 (period circadian protein homolog 1/2), or RARα (retinoic acid receptor alpha) [50]. Increased CMA activity is usually related to lipotoxicity (cytosolic lipid overload), starvation, hypoxia, and mitochondrial or ER stress. The elevated CMA activity is affected by the transcriptional upregulation of LAMP-2A (mostly by NFE2L2 (NFE2-Like BZIP transcription factor 2), Nrf2, and NFAT1 (nuclear factor of activated T cells) [51].

The CMA pathway supports a range of cellular processes, including the maintenance of protein quality, the regulation of the cell cycle, and the modulation of immune responses. Especially, CMA contributes to the quality control of mitochondrial proteins, such as COX IV (cytochrome c oxidase subunit 4), Tom20 (mitochondrial import receptor subunit TOM20 homolog), DJ-1 (nucleic acid deglycase), Parkin, MFN2, ATP5F1A (ATP synthase F1 subunit alpha), and VDAC1, which modulate mitochondrial function and protect the integrity of mitochondria [45].

## 3. Role of Autophagy in Tumorigenesis

In cancer, the autophagy process serves a dual and complex function, acting either as a suppressor or initiator of tumorigenesis, depending on the type of tumor and the stage of disease advancement. On the one hand, autophagy might promote cancer suppression by abolishing oxidative stress, inhibiting cellular transformation, preventing the accumulation of damaged cellular components, and maintaining cell homeostasis [11]. Transcription factors like p53, death-associated protein kinase (DAPK), tuberous sclerosis proteins 1 and 2 (TSC1/2), and phosphatase and tensin homolog (PTEN) are essential contributors to the tumor-suppressive function of autophagy [11].

On the other hand, autophagy can support tumor development and metastasis by providing nutrients to cancer cells [52], allowing cancer cells to survive under metabolic stress. Tumor oncogenes, like RAS and BRAF, promote tumor growth by upregulating the process of autophagy [11]. In developing novel cancer therapies or enhancing the efficacy of chemotherapy, comprehending the involvement of autophagy at all phases of tumor formation is crucial. This knowledge contributes to the advancement of precision therapies that can effectively modulate autophagy [53].

The involvement of the autophagy activator Beclin-1 in tumorigenesis is linked to its phosphorylation level and interactions with various partner proteins [54]. DAPK is a key regulator protein that contributes to the phosphorylation status of Beclin-1 and the formation of the autophagosome [55]. Autophagy inhibition is achieved through the interaction of Beclin-1 with its inhibitor, BCL2. In contrast, the interaction of Beclin-1 with AMBRA1, UVRAG (UV-radiation-resistance-associated gene protein), and BIF1 (Bax interacting factor 1) disrupts the binding of Beclin-1 with BCL2, which subsequently leads to autophagy initiation [11]. In solid tumors, a decrease in Beclin-1 expression is often observed and correlated with metastasis development. On other hand, in colorectal cancers and gastric carcinomas, Beclin-1 expression is elevated, resulting in enhanced autophagy. This observation has led to the suggestion that Beclin-1 promotes cell proliferation and tumorigenesis under stress conditions like hypoxia and starvation [56]. Furthermore, Beclin-1 cooperates with another autophagy regulator, PTEN, which negatively controls the activity of the PI3K/AKT pathway. A reduced expression of PTEN and Beclin-1 has been observed in chemoresistant ovarian cancers [57]. This discovery indicates that PTEN and Beclin-1 play roles in regulating autophagy in ovarian cancer, and their decreased expression levels contribute to reducing autophagy activity and increasing chemoresistance [58].

The modulation of autophagy by the major transcription factor p53 is determined by its subcellular localization, which determines whether cancer cells will survive or die. Nuclear p53 facilitates autophagy activation by promoting the transcription of autophagy-related genes, while cytoplasmic p53 suppresses autophagy by inhibiting autophagy regulators [11]. Upon encountering cellular stress, nuclear p53 initiates autophagy through inducing the expression of DRAM (damage-regulated autophagy modulator), DAPK, and ULK1/2 [59]. Moreover, p53, through the promotion of autophagy, can increase the proliferation and resistance to chemotherapy of malignant liposarcoma cells [60].

The accumulation of autophagy cargo receptor p62 (also known as SQSTM1) is a notable feature observed in many cancers, and it is correlated with poor clinical outcomes among hepatocellular carcinoma patients [61] and increased metastasis occurrence in nasopharyngeal carcinoma. The accumulation of p62 supports tumor development and cancer cell growth via the activation of Nrf2, mTORC1, TRAF6 (tumor-necrosis-factor-receptor-associated factor 6), TNFa (tumor necrosis factor α), and NF-kB (nuclear factor kappa-light-chain-enhancer of activated B cells) [54]. Consequently, an increased presence of p62 in tumors implies its involvement in promoting the development and progression of cancer. Therefore, inhibiting p62 during autophagy holds promise as a strategy for treating cancer [62].

Among mitophagy regulators, it has been observed that the tumor suppression Parkin protein encoded by the *PARK2* gene is frequently deleted in colorectal, lung, breast, glioblastoma, and melanoma cancers. The absence of Parkin E3 ubiquitin ligase leads to the accumulation of dysfunctional mitochondria, resulting in elevated levels of glycolysis and ROS, reduced OXPHOS, and increased resistance to apoptosis in cells [22].

The mitochondrial kinase PINK1 is implicated in tumor suppression due to its role in detecting and removing damaged mitochondria. In certain cancers, there is a noted decrease in PINK1 expression (such as sarcomas, neuroblastomas, and leukemias), while in others, there is an increase (such as lung and breast cancers and carcinoma) [63].

Autophagy has been demonstrated to play a pivotal role in promoting drug resistance in chemotherapy-treated cancer cells. It also regulates cell migration and metastasis by affecting the interactions between cancer and healthy cells [11]. Despite the complex role of autophagy in cancer, inhibiting this process can make cancer cells more sensitive to chemotherapy and enhance cell death [24].

## 4. Modulation of Mitochondria and Autophagy Exhibits Promise in Cancer Treatment

Cancer cells frequently demonstrate the capacity to reprogram their metabolism, allowing them to survive and thrive in challenging conditions, including those generated by chemotherapy [6]. Targeting metabolic plasticity in cancer has been shown to significantly enhance the effectiveness of cancer therapies [64]. A recent study has shown that decreased expression of mitochondrial fission regulator protein DRP1 affects metabolic plasticity and reduces the survival of breast-cancer--induced brain metastases [65].

Mitochondria primarily drive the bioenergetic adaptation that facilitates tumor growth. It is well established that mitochondrial reprogramming promotes tumor growth and cancer cell proliferation via retrograde signaling involving ROS, Ca^2+^, ATP, or TCA intermediates, which can modify gene expression [6]. It has recently been identified that the knockout of *MTCH1* (*mitochondrial carrier 1*) in cervical cancer (in HeLa cells) activates retrograde signaling through the FOXO1-GPX4 axis, leading to increased accumulation of mtROS and ferroptosis. The study in question proposes the use of MTCH1 as a candidate target for retrograde signaling pathways in cervical cancer [66].

Modifying mitochondrial metabolism (Table 2 and Table 3) offers a strategy for reshaping cancer cell metabolism and combatting drug resistance. Current strategies for targeting mitochondrial function include inhibiting ETC, modulating redox balance, affecting Ca^2+^ homeostasis or the apoptotic pathway, and disrupting the TCA cycle. Disruption of ETC can be achieved through the inhibition of ETC complexes I–V. The effectiveness of several complex I inhibitors is limited by issues like poor potency, toxicity, or unintended off-target actions, such as targeting rotenone and BAY 87-2243 [67]. While some complex I inhibitors have failed to translate successfully to preclinical studies, others are currently being tested in clinical trials (Table 3). Phenformin, an antidiabetic drug, inhibits complex I and disrupts the redox balance (NADH/NAD+) and energetic state (AMP/ATP), resulting in AMPK activation [68]. Atovaquone, used as an antimalarial drug, interferes with complex III, reducing oxygen consumption and subsequently decreasing tumor hypoxia in individuals with non-small-cell lung cancer [69].

Through the excessive activation of mitochondrial respiration, the loss of MMP can cause a breakdown in mitochondrial metabolism and ATP production, resulting in cell death. A newly identified complex IV activator, the fungal natural product ophiobolin A (OPA), significantly decreases NCI-H1703 cells’ proliferation [70]. Bedaquiline interferes with ATP production by targeting complex V, which lowers DA-MB-231 breast cancer cell proliferation, enhances ovarian cancer cells’ sensitivity to cisplatin, and helped prevent metastasis in a xenograft model [71,72].

The disruption of mitochondrial metabolism through TCA cycle inhibition using devimistat (CPI-613), PDH, and KGDH inhibitors is being tested in patients with advanced biliary tract cancer in vitro and in a Phase Ib clinical trial, in combination with gemcitabine and cisplatin [73]. Current clinical trials are investigating devimistat (CPI-613) as a monotherapy for refractory Burkitt’s lymphoma/leukemia (NCT03793140) and in combination with chemotherapies for advanced pancreatic cancer (NCT03699319) and with chemoradiation for pancreatic adenocarcinoma (NCT05325281) (Table 3).

Inhibiting the apoptosis regulator BCL-2 (B-cell lymphoma-2) is one of the most extensively investigated approaches for triggering the mitochondrial apoptotic pathway in cancer therapy. Venetoclax (ABT199) was the first FDA-approved BH3-mimetic drug, originally indicated for the treatment of chronic lymphocytic leukemia [74]. Subsequent studies revealed its effectiveness in treating acute myeloid leukemia. It is commonly administered either as monotherapy or in combination with monoclonal antibodies, such as rituximab, or alongside chemotherapy. The molecular action of ventoclax is driven by the activation of BAK and BAX proteins, leading to the permeabilization of the mitochondrial outer membrane and inducing apoptosis [75]. Ongoing preclinical and clinical (Table 3) studies are investigating the efficacy and safety of venetoclax, both as a monotherapy and in combination with other anti-cancer drugs, in treating breast cancer (NCT03900884), myeloma (NCT05455294), lung cancer (NCT04274907), prostate cancer (NCT03751436), and solid tumors [76].

Alongside its mitochondrial function, autophagy helps facilitate cancer plasticity under nutrient deprivation conditions. Inducing autophagy can potentially prevent tumor development and growth in the early stages [77]. However, in advanced stages of cancer, autophagy supports tumor growth and metastasis by supplying the necessary substrates for cell proliferation [78]. The impact of autophagy activation on tumorigenesis is influenced by the degree of autophagy. A basic level of autophagy facilitates tumor growth and the development of drug resistance, while a high level of autophagy results in excessive removal of cellular components, leading to the cell death [79]. Relying only on autophagy targeting is insufficient for cancer treatment. Some studies have demonstrated that combining autophagy inhibitors or activators with chemotherapy, radiotherapy, or immunotherapy is a more effective treatment strategy [77]. By inhibiting autophagy, the susceptibility of cancer cells to chemotherapeutic drugs and treatments that induce apoptosis is heightened [78]. Table 2 and Table 3 present a range of autophagy inhibitors and activators that are being investigated in preclinical studies and ongoing clinical trials. As FDA-approved antimalarial drugs, chloroquine (CQ) and hydroxychloroquine (HCQ) are some of the most prominent autophagy inhibitors being explored in cancer therapies based on autophagy mechanisms. Through their accumulation in lysosomes and inhibition of lysosomal acidification, they interrupt the autophagosome’s fusion with lysosome and change signaling and transcriptional activity [80]. The antitumor effect of HCQ was increased when used in combination with monoclonal antibodies, namely, anti-PD1 (nivolumab), in advanced melanoma (Table 3, NCT04464759); inhibitor of MEK1/2 (trametinib) in pancreatic cancer (NCT03825289); a Ras/Raf/MEK/ERK signaling pathway inhibitor (sorafenib) in hepatocellular cancer (NCT03037437); and an Akt inhibitor (MK2206) in solid tumors (NCT01480154). CQ and HCQ, in addition to changing lysosomal pH, also impact the pH values of Golgi vesicles and endosomes [81]. CQ derivatives are also known to target and suppress the function of PPT1 (palmitoyl-protein thioesterase 1) in melanoma cells [82]. The precise mechanisms of action of CQ and HCQ remain poorly understood and extend beyond their effects on autophagy. It has been observed that CQ activates the p53 pathway, resulting in apoptosis in glioma cells [83]. In clinical trials, adverse events associated with CQ and HCQ, including nausea, diarrhea, vomiting, myopathy, and cardiotoxic effects, are frequently reported [84].

Conversely, stimulating autophagy in cancer treatment can effectively impede cell proliferation and inhibit tumor growth. Among the leading autophagy inducers examined in clinical trials are the FDA-approved mTOR inhibitors rapamycin (sirolimus) and its analogue temsirolimus (CCI-779) and everolimus, which are FDA-approved for the treatment of malignancies and the prevention of transplant rejection [85]. Everolimus and temsirolimus are FDA-approved drugs used to treat advanced renal cell carcinoma. Ongoing research, including preclinical and clinical trials, is exploring their efficacy as a monotherapy and in combination with other therapies (CQ, radiation, and THZ1 (cyclin-dependent kinase 7 inhibitor)) across different cancer types (bladder, colorectal, and prostate cancers and carcinoma) in vitro and in vivo. Research is focusing on everolimus, both as a standalone treatment and in combination with CQ, HCQ, AKT inhibitors 1 and 2, arsenic trioxide, or propachlor, in breast, renal, and ovarian cancer cells; various carcinoma cell lines; and mouse models. Investigations have been conducted on rapamycin in cell lines of pancreatic, cervical, and lung carcinomas; melanomas; osteosarcomas; and liposarcomas, as well as in xenograft mice models [86]. Clinical trials have explored the use of rapamycin in combination with chloroquine (CQ) and hydroxychloroquine (HCQ). Current ongoing research includes a Phase I trial assessing rapamycin’s combination with vorinostat for treating advanced cancers (NCT01087554), as well as a Phase I/II study evaluating its use with HCQ, metformin, dasatinib, or nelfinavir in treating relapsed prostate cancer and other solid tumors (NCT05036226).

Recently, the natural polyphenol epigallocatechin gallate (EGCG) deriving from green tea leaves has garnered interest for its ability to induce cell death through autophagy and apoptosis. EGCG is known to alter multiple cellular pathways in different types of cancer. Among the most notable pathways it effects is the RAS-Raf-MEK-ERK axis, where its action leads to the inhibition of cell proliferation and the induction of apoptosis in pancreatic cancer [87,88]. Moreover, EGCG attenuated the PTEN/AKT/mTOR pathway in ovarian cancer cell lines and mouse models [89]. In bladder cancer cell lines (5634 and T24), EGCG induces apoptosis through the regulation of autophagy in a dose-dependent manner. EGCG upregulates the expression of caspase 3, caspase 9, and Bax, and it also decreases BCL2 expression. Concurrently, EGCG stimulates the formation of autophagosomes and elevates the expression of the autophagy-related protein LC3II [90].

**Table 2:** Synthetic inducers and inhibitors of mitochondrial function and autophagy.

Compound	Mechanism	Ref.
Mitochondria inhibitors		
Rotenone	Inhibits complex I in gastric cancer cells (MKN-1, MKN-B, and MKN-74)	[91]
BAY 87-2243	Inhibited complex I in melanoma tumor xenograft (SK-MEL-28 and G-361 cells) and in vitro (A-375, G-361, SK-MEL-5, SK-MEL-28 cells)	[92]
MitoVES (mitochondrially targeted vitamin E succinate)	Inhibits complex II in colon cancer (HCT116 cells in vitro and in BALB/c nu/nu mice)	[93]
Atovaquone (ATO)	Inhibits complex III in breast cancer cells (MCF7)	[94]
Antimycin A	Inhibits complex III in acute myeloid leukemia U937 and HL-60 cells	[95]
VLX600	Acts as an OXPHOS inhibitor (inhibiting complexes I, II, IV) in colon cancer 3-D microtissues; Acts as an iron chelator; Induces autophagy-dependent cell death and mitophagy through BNIP3/BNIP3L activation in glioblastoma cells (U251, MZ54, NCH644) and organotypic brain slice cultures	[96]
BTB06584	Inhibits ATP synthase in non-small-cell lung cancer cells (A549)	[97]
Oligomycin	Inhibits ATP synthase in breast metastasis cells (MDA-MB-231)	[98]
Mitochondrial activators		
Ophinobolin A (OPA)	Activates complex IV in lung squamous cell carcinoma (NCI-H1703)	[70]
Autophagy inhibitors		
Vitexin	Inhibits LC3-associated autophagosome formation Decreases p-ERK1/2 levels in hepatocellular carcinoma (SK-Hep1 and Hepa1-6 cells)	[99]
RA-XII	Inhibits AMPK pathway; Induces apoptosis through the suppression of autophagy in liver cancer (HepG2 cells);	[100]
3-methyladenine (3-MA)	Inhibits LC3-I/II and class III PI3K complex; Increases p62 levels in colon cancer cells (LOVO and SW480)	[101]
Astragaloside II	Decreases the levels of LC3-II and Beclin-1 in hepatic cancer cell lines (Bel-7402 and Bel-7402/FU)	[102]
Bafilomycin A1	Increases LC3 levels; Promotes the association of Beclin-1 and Bcl-2; Blocks V-ATPase in B-cell acute lymphoblastic leukemia (697 cells)	[103]
SAR405	Suppresses VPS34 and PIK3C3 kinase in carcinoma cell line H1299	[104]
Clarithromycin	Upregulates LC3-II in primary colorectal cancer surgical samples; Induces autophagosome formation and decreases p62/SQSTM1 levels in colorectal cancer (HCT116 cells)	[105]
4-acetylantroquinonol B	Inhibits ATG5 in ovarian cell line ES-2	[106]
Autophagy activators		
Salinomycin	Increases LC3B-II levels and vacuolization in melanoma SK-Mel-19 cells	[107]
Esomeprazole	Inhibits V-ATPase in lung cancer (A549/Taxol cells)	[108]
Niraparib	Inhibits AKT/mTOR pathway and increases ROS levels; Activates ERK1/2 and increases LC3-II in hepatocellular carcinoma (Huh7 and HepG2)	[109]
Matrine	Leads to the accumulation of LC3-II; Reduces the levels of total AKT and mTOR in gastric cancer (SGC-7901)	[110]
Bisindolylmaleimide (BMA-155Cl)	Increases Beclin-1, NF-kB, and p65 levels in hepatocarcinoma HepG-2 cells	[111]
Resveratrol Spermidine	Activates SIRT1 in colon cancer HCT 116 cells	[112]
Bicyclol	Inhibits p-AKT and pERK; Decreases the levels of p-mTOR (Ser2448); Increases LC3-II levels in hepatocellular carcinoma cell HepG2	[113]
Glycochenodeoxycholate	Increases LC3-II and pAMPK levels; Decreases p63 and pmTOR levels in hepatocellular carcinoma (SMMC7721 and Huh7 cells)	[114]
Lapatinib	Increases LC3-II, ATG7, Beclin-1, and ATG5 levels in acute myeloblastic leukemia (U937 cells)	[115]
Lycorine	Decreases TCRP1 (tongue-cancer-resistance-associated protein 1) and p-AKT levels, increases LC3 II levels, and decreases Beclin-1 levels in hepatocellular carcinoma (HepG2 and SMMC-7721)	[116]
Baicalein/baicalin	Activates ATG5, ATG7, ATG12, Beclin-1, and LC3-IIB proteins in bladder cancer T24 cells	[117]
Epigallocatechin gallate (EGCG)	Elevates levels of LC3-II; Increases number of autophagosomes in cepatocellular carcinoma cell line HepG2	[118]

**Table 3:** Investigated drugs targeting autophagy and mitochondrial pathways in ongoing cancer clinical trials.

Title	Drug	Target	Cancer Type	ClinicalTrials.gov ID	Ref.
Targeted apoptotic pathway					
A Phase I clinical trial evaluating the tolerance and pharmacokinetics of TQB3909 tablets in patients with relapsed or refractory advanced malignant tumors (China)	TQB3909	BCL-2 inhibitor	Phase I: advanced malignant tumors	NCT04975204	[119]
A Phase Ib/ii study to investigate the safety, tolerance and pharmacokinetics of TQB3909 with HR-positive, HER2-negative advanced breast cancer (China)			Phase Ib/II: advanced breast cancer	NCT05775575	
A Phase Ib/II clinical trial on the safety and efficacy of TQB3909 tablets in patients with recurrent or refractory CLL/SLL (China)			Phase Ib/II: chronic lymphocytic leukemia/small lymphocytic lymphoma	NCT05959694	
A Phase 1a/1b open-label dose escalation and expansion study of Bcl-2 inhibitor BGB-11417 in patients with mature B-cell malignancies (United States)	Sonrotoclax (BGB-11417) or Sontroclax in combination with zanubrutinib and obinutuzumab	BCL-2 inhibitor	Phase Ia/Ib: mature B-cell malignancies	NCT04277637	[120]
A Phase I study of venetoclax in combination with cytotoxic chemotherapy, including calaspargase pegol, for children, adolescents and young adults with high-risk hematologic malignancies (United States)	Venetoclax in combination with azacitidine cytarabine, methotrexate, hydrocortisone, leucovorin, dexamethasone, vincristine, doxorubicin, dexrazoxane, calaspargase, pegol, erwinia asparaginase		Acute myeloid leukemia/chronic lymphocytic leukemia Phase I: hematologic malignancies	FDA-approved NCT05292664	
A Phase 1 study of triplet therapy with navitoclax, venetoclax, and decitabine for high-risk myeloid malignancies (United States)	Venetoclax in combination with navitoclax and decitabine		Phase I: myeloid malignancy	NCT05455294	
Phase 1 study of venetoclax, a BCL2 antagonist, for patients with blastic plasmacytoid dendritic cell neoplasm (BPDCN) (United States)	Venetoclax		Phase I: dendritic cell neoplasm	NCT03485547	
A Phase 1b study of palbociclib, letrozole and venetoclax in ER and BCL-2 positive locally advanced or metastatic breast cancer (Australia)	Venetoclax in combination with palbociclib and letrozole		Phase Ib: breast cancer	NCT03900884	
Phase Ib/II study of enzalutamide with venetoclax (ABT-199) in patients with metastatic castrate resistant prostate cancer (mCRPC) (United States)	Venetoclax in combination with enzalutamide		Phase Ib/II: prostate cancer	NCT03751436	
A Phase 1b study of venetoclax in combination with pembrolizumab in subjects with previously untreated NSCLC whose tumors have high PD-L1 expression (United States)	Venetoclax in combination with pembrolizumab		Phase Ib: non-small-cell lung cancer		
A Phase 1 study of oral LOXO-338, a selective BCL-2 inhibitor, in patients with advanced hematologic malignancies (United States)	LOXO-338		Phase I: advanced hematologic malignancies	NCT05024045	[121]
A Phase 1 study investigating the safety, tolerability, pharmacokinetics, pharmacodynamics, and preliminary antitumor activity of second mitochondrial-derived activator of caspases mimetic BGB-24714 as monotherapy and with combination therapies in patients with solid tumors	BGB-24714 or BGB-24714 in combination with paclitaxel, carboplatin, docetaxel	SMAC (mitochondrial-derived activator of caspases) mimetic and inhibitor of apoptosis protein	Phase I: solid tumors	NCT05381909	[122]
A randomized, double-blind placebo-controlled, Phase 3 study of Debio 1143 in combination with platinum-based chemotherapy and standard fractionation intensity-modulated radiotherapy in patients with locally advanced squamous cell carcinoma of the head and neck, suitable for definitive chemoradiotherapy (TrilynX) (United States)	Xevinapant (Debio 1143) in combination with chemotherapy	Second mitochondrial-derived activator of caspases	Phase III: advanced squamous cell carcinoma of the head and neck	NCT04459715	[123]
A Phase 1b study of the OxPhos inhibitor ME-344 combined with bevacizumab in previously treated metastatic colorectal cancer (United States)	Me-344 combined with bevacizumab	OxPhos pathway inhibitor; purine biosynthesis inhibitor	Phase Ib: previously treated metastatic colorectal cancer	NCT05824559	[124]
A Phase 1 open-label, dose-escalation, safety, pharmacokinetic, and pharmacodynamic study of Minnelide™ capsules given alone or in combination with paclitaxel in patients with advanced gastric cancer (Republic of Korea)	Minnelide (triptolide)	SIRT3 regulator; c-myc down-regulator	Phase I: gastric cancer	NCT05566834	[125]
A Phase II trial of the superenhancer inhibitor minnelide in advanced refractory adenosquamous carcinoma of the pancreas (ASCP) (United States)			Phase II: advanced refractory adenosquamous carcinoma of the pancreas	NCT04896073	
A Phase 1b open-label, dose-escalation, safety, and pharmacodynamic study of Minnelide™ capsules given in combination with osimertinib in patients with EGFR mutated NSCLC (United States)	Minnelide in combination with osimertinib		Phase Ib: lung cancer	NCT05166616	
A Phase 1b, open-label, safety, pharmacokinetic, and pharmacodynamic study of an anti-super-enhancer Minnelide given along with abraxane plus gemcitabine in patients with metastatic adenocarcinoma of the pancreas (Republic of Korea)	Minnelide in combination with Abraxane and gemcitabine		Phase Ib: metastatic adenocarcinoma of the pancreas	NCT05557851	
A Phase 1, multi-center, open-label, dose-escalation, safety, pharmacokinetic, and pharmacodynamic study of minnelide™ capsules given alone or in combination with protein-bound paclitaxel in patients with advanced solid tumors (United States)	Minnelide in combination with paclitaxel		Phase I: advanced solid tumors	NCT03129139	
Targeting mitochondrial metabolism					
A Phase I trial targeting mitochondrial metabolism with papaverine in combination with chemoradiation for stage II-III non-small cell lung cancer (United States)	Papaverine in combination with chemoradiation and immunotherapy	Mitochondrial Complex I inhibitor	Phase I: Stage II–III non-small-cell lung cancer	NCT05136846	[126]
Phase I trial of phenformin with patients with combination BRAF inhibitor/MEK inhibitor in patients with BRAFV600E/K-mutated melanoma (United States)	Phenformin in combination with dabrafenib and phenformin	Mitochondrial complex I inhibitor	Phase I: melanoma	NCT03026517	[68]
Phase II clinical trial repurposing atovaquone for the treatment of platinum-resistant ovarian cancer (United States)	Atovaquone (Mepron)	Mitochondrial complex III inhibitor	Phase II: ovarian cancer	NCT05998135	[127]
A Trial of atovaquone (Mepron^®^) combined with conventional chemotherapy for de novo acute myeloid leukemia (AML) adolescents, and young adults (ATACC AML) (United States)	Atovaquone in combination with conventional chemotherapy (cytarabine, daunorubicin, etoposide, gemtuzumab ozogamicin)	Mitochondrial complex III inhibitor	Phase I: acute myeloid leukemia	NCT03568994	
A Phase I study of oral carboxyamidotriazole orotate (CTO) titrated as a single agent in patients with advanced or metastatic solid tumors and titrated in combination therapy with temodar^®^ for patients with glioblastoma and other recurrent malignant gliomas or in combination with temodar^®^ and radiation therapy for patients with newly diagnosed glioblastoma and malignant gliomas (United States)	Carboxyamidotriazole orotate or in combination with temodar/radiation therapy	Non-voltage-dependent calcium channel inhibitor	Phase I: advanced or metastatic solid tumors Phase I: glioblastoma, malignant gliomas	NCT01107522	[128]
A Phase II clinical trial of CPI-613 in patients with relapsed or refractory Burkitt lymphoma/leukemia or high-grade B-cell lymphoma with rearrangements of MYC and BCL2 and/or BCL6 (United States)	Devimistat (CPI-613)	Pyruvate dehydrogenase and α-ketoglutarate dehydrogenase/2-oxoglutarate dehydrogenase	Phase II: relapsed/refractory Burkitt’s Lymphoma/leukemia or high-grade B-cell lymphoma with rearrangements of MYC and BCL2 and/or BCL6	NCT03793140	[129]
A Phase II/I open-label clinical trial of CPI-613 in combination with modified FOLFIRINOX in patients with locally advanced pancreatic cancer and good performance status (United States)	Devimistat in combination with modified FOLFIRINOX (oxaliplatin, irinotecan, 5-flurouracil, and folinic acid)		Phase II/I: advanced pancreatic cancer	NCT03699319	
A Phase I dose-escalation study of CPI-613 (Devimistat) in combination with chemoradiation in patients with pancreatic adenocarcinoma (United States)	Devimistat in combination with chemoradiation		Phase I: pancreatic adenocarcinoma	NCT05325281	
Phase II open-label multi-cohort study evaluating CPI-613 (Devimistat) in combination with hydroxychloroquine and 5-fluorouracil or gemcitabine in patients with advanced chemorefractory colorectal, pancreatic, or other solid cancers (United States)	Devimistat in combination with hydroxychloroquine 5-fluorouracil or gemcitabine		Phase II: advanced chemorefractory colorectal, pancreatic or solid tumors	NCT05733000	
Phase II study of AG-120 in people with IDH1 mutant chondrosarcoma (United States)	Ivosidenib (AG-120)	IDH1 inhibitor	Acute myeloid leukemia Phase II: Chondrosarcoma	FDA-approved NCT04278781	[130]
A Phase I, multicenter, open-label, dose-escalation and expansion, safety, pharmacokinetic, pharmacodynamic, and clinical activity study of orally administered AG-120 in subjects with advanced hematologic malignancies with an IDH1 mutation (United States)			Phase I: advanced hematologic malignancies	NCT02074839	
Phase Ib/II investigator initiated study of the IDH1-mutant inhibitor ivosidenib (AG120) with the BCL2 inhibitor venetoclax +/- azacitidine in IDH1-mutated hematologic malignancies (United States)	Ivosidenib in combination with venetoclax +/- azacitidine		Phase Ib/II: IDH1-mutated hematologic malignancies	NCT03471260	
Phase II study of enasidenib in IDH2-mutated malignant sinonasal and skull base tumors (United States)	Enasidenib	IDH2 inhibitor	FDA-approved for acute myeloid leukemia Phase II: malignant sinonasal and skull base tumors	NCT06176989	[131]
Trial of dichloroacetate (DCA) in glioblastoma multiforme (GBM) (United States)	Dichloracetate	Pyruvate dehydrogenase complex inhibitor	Phase IIA: glioblastoma	NCT05120284	[132]
Targeting autophagy					
LIMIT melanoma: (lysosomal inhibition + melanoma immunotherapy) a Phase 1/2 open label trial of nivolumab and hydroxychloroquine or nivolumab/ipilimumab and hydroxychloroquine in patients with advanced melanoma (United States)	Hydroxychloroquine in combination with nivolumab/ipilimumab	Lysosomal acidification inhibitor; Disrupt the fusion of autophagosome with lysosome	Phase I/II: melanoma	NCT04464759	[81,133]
THREAD: A Phase I trial of trametinib and hydroxychloroquine in patients with advanced pancreatic cancer (United States)	Hydroxychloroquine in combination with trametinib		Phase I: advanced pancreatic cancer	NCT03825289	
Binimetinib plus hydroxychloroquine in KRAS mutant metastatic pancreatic cancer (United States)	Hydroxychloroquine in combination with binimetinib		Phase I: KRAS mutant metastatic pancreatic cancer	NCT04132505	
Modulation of sorafenib induced autophagy using hydroxychloroquine in hepatocellular cancer (United States)	Hydroxychloroquine in combination with sorafenib		Phase II: advanced hepatocellular cancer	NCT03037437	
A Phase I trial of MK-2206 and hydroxychloroquine in solid tumors, melanoma, renal and prostate cancer to examine the role of autophagy in tumorigenesis (United States)	Hydroxychloroquine in combination with Akt inhibitor MK2206		Phase I: advanced solid tumors, melanoma, prostate, kidney cancer	NCT01480154	
Treatment of adults with newly diagnosed glioblastoma with partial brain radiation therapy plus temozolomide and chloroquine followed by tumor treating fields plus temozolomide and chloroquine—a pilot study (United States)	Chloroquine in combination with radiotherapy or tumor-treating fields therapy		Phase I: glioblastoma	NCT04397679	
Phase II study of oral metformin for intravesical treatment of non-muscle-invasive bladder cancer (Netherlands)	Metformin	AMPK activator; mTOR inhibitor; STAT3-mediated pathway inhibitor; autophagy inducer (decreases p62, increases LC3-II); Complex I inhibitor	Phase II: non-muscle-invasive bladder cancer	NCT03379909	[134,135,136]
STOP-LEUKEMIA: Repurposing metformin as a leukemia-preventive drug in CCUS and LR-MDS (Denmark)	Metformin		Phase II: clonal cytopenia, myelodysplastic neoplasms	NCT04741945	
Clinical effects of metformin on fertility-sparing treatment for early endometrial cancer (Republic of Korea)			Phase III: endometrial cancer	NCT04792749	
Profiling and reversing metabolic insufficiency in the tumor microenvironment in advanced melanoma: a trial of pembrolizumab and metformin versus pembrolizumab alone in advanced melanoma (United States)	Metformin in combination with pembrolizumab		Phase I: advanced melanoma	NCT03311308	
Phase 2A pilot trial of metformin, digoxin, simvastatin (C3) in combination with gemcitabine in subjects with recurrent/refractory metastatic advanced pancreatic cancer) (United States)	Metformin in combination with simvastatin, and digoxin +/- gemcitabine		Phase I/II: metastatic advanced pancreatic cancer	NCT06030622	
Effect of metformin plus tyrosine kinase inhibitors compared with tyrosine kinase inhibitors alone for patients with advanced non-small cell lung cancer and EGFR mutations: Phase 3 randomized clinical trial (Mexico)	Metformin in combination with tyrosine kinase inhibitors		Phase II: advanced non-small-cell lung cancer	NCT05445791	
A Phase 0, single-center, open-label, dose-escalating trial using super-selective intra-arterial infusion of a single dose of temsirolimus for the treatment of recurrent high-grade glioma (United states)	Temsirolimus (CCI-779)	Autophagy in-ducer; mTOR inhibitor	Advanced renal cell carcinoma Early phase 0: glioma, glioblastoma	FDA-approved NCT05773326	[137]
Phase II trial of encapsulated rapamycin (eRapa) for bladder cancer prevention (United States)	Rapamycin (Sirolimus)	Autophagy inducer; mTOR inhibitor	Phase II: bladder cancer	NCT04375813	[138]
A Phase I trial of sirolimus or everolimus or temsirolimus (mTOR inhibitor) and vorinostat (histone deacetylase inhibitor) in advanced cancer (United States)	Rapamycin in combination with vorinostat		Phase I: advanced cancer	NCT01087554	
Combination of autophagy selective therapeutics (COAST) in advanced solid tumors or relapsed prostate cancer, a Phase I/II Trial (United States)	Rapamycin in combination with hydroxychloroquine, metformin or dasatanib or nelfinavir		Phase I/II: advanced solid tumors, relapsed prostate cancer	NCT05036226	
	Everolimus (afinitor)	Autophagy in-ducer; mTOR inhibitor	HER2-negative advanced breast cancer, pancreatic neuroendocrine tumors, renal cell carcinoma, angiomyolipoma	FDA-approved	[139,140]
Efficacy and safety of epigallocatechin-3-gallate, an important polyphenolic that originates from tea, in patients with esophageal squamous cancer: a Phase II trial (China)	Epigallocatechin gallate (EGCG)	Autophagy activator through ROS elevation, Beclin-1- and LC3B-increasing	Phase II: esophageal squamous cancer	NCT06398405	[87]

## 5. Challenges Faced in Relation to Therapies Targeting Mitochondrial and Autophagic Processes

Utilizing autophagy and mitochondrial function modulators (Table 2 and Table 3) in cancer therapy shows potential in overcoming tumor plasticity and drug resistance. However, some compounds exhibit dual effects, including non-specificity and undefined molecular mechanisms of action. For instance, EGCG activates autophagy and has been shown to suppress COX-2 in prostate, colon, and skin cancers in vitro and in mouse models as well as inhibit NF-kB in a melanoma mouse model [141]. In colorectal cancer (the HT-29 cell line), it triggers endoplasmic reticulum stress through the upregulation of BiP and PERK, leading to apoptosis via increased caspase-3/7 levels. In glioblastoma (the T98G and U87MG cell lines), EGCG elevates ROS levels, increases caspase 8 levels, and activates the JNK pathway [142]. Metformin is an exemplary mitochondria-targeted drug, as it decreases TCA cycle activity and inhibits complex I, leading to reduced ATP production. The resulting lower ATP levels activate AMPK and inhibit mTOR, which triggered autophagy in a myeloma cancer model (the RPMI8226 and U266 cell lines and NOD/SCID mice). In contrast, in leukemic cells (HL60 and MOLM14), metformin triggers apoptosis. Additionally, metformin has the ability to inhibit the NF-kB signaling pathway [143]. These examples underscore the pressing need to develop drugs that specifically target autophagy or mitochondria.

The development of autophagy inhibition methods centers on either inducing excessive autophagy or targeting the early stages of autophagy initiation [59]. A potential candidate for targeting autophagy initiation, the ULK1 inhibitor 13-oxyingenol-dodecanoate (13OD), is currently undergoing preclinical research. The associated study demonstrated that 13OD effectively inhibited the proliferation of non-small-cell lung cancer cells (A549 and H460) in vitro and in BALB/c athymic nude mice by promoting autophagic cell death [144].

Autophagy inducers, notably mTOR inhibitors, face challenges due to their incomplete targeting of mTORC1. This limitation arises as mTORC1 can bypass rapalog effects through the compensatory activation of other pathways, such as PI3K/Akt. Additionally, mutations in the FKBP12–rapamycin binding domain, including an alanine-to-valine substitution at position 2034 (A2034V) and a phenylalanine-to-leucine substitution at position 2108 (F2108L), as well as the activation of mTORC2-dependent pathways, contribute to the issue [145].

Following clinical trials, it was revealed that HCQ’s therapeutic effects are not mainly induced by autophagy inhibition. Rather, HCQ accumulates in endosomes, inhibits the toll-like receptor (TLR) pathway, reduces self-antigen presentation, and curbs cytokine production. The acidic nature of the tumor microenvironment negatively impacts HCQ’s efficacy by restricting its cellular transport. To address this challenge and minimize toxicity, targeted drug delivery systems like nanoparticles can be utilized [146].

Researchers conducting clinical trials struggle with the challenge of identifying which cancer types and grades are autophagy-dependent, requiring them to discern the function of autophagy each specific cancer patient [59]. To tackle the issue of identifying cancer’s dependence on autophagy, some clinical studies employ biomarkers. A case in point is a clinical trial that evaluated glioblastoma patients’ responses to combined CQ, chemotherapy, or radiotherapy by analyzing the EGFRvIII marker [147]. The level of the autophagy marker p62 is influenced not only by autophagy activity but also by its role in activating antioxidant gene expression, particularly NRF2, even when autophagy is not occurring [59].

For the mitochondria-targeted BCL-2 inhibitor venetoclax, clinical trials have revealed that secondary resistance can arise in multiple myeloma patients who have undergone long-term venetoclax therapy or possess missense mutations in BCL-2 and BAX. Venetoclax treatment, whether administered as a monotherapy or in combination with chemotherapy, can cause adverse events, including nausea, diarrhea, tumor lysis syndrome, and, most commonly, neutropenia and thrombocytopenia [148]. Studies investigating IDH1/IDH2 inhibitors indicate that resistance can occur in solid malignancies. It is hypothesized that this resistance arises from isotype switching, wherein patients with cytosolic IDH1 mutations develop mitochondrial IDH2 mutations after receiving IDH1 inhibitor therapy [149].

Despite their potential, the application of autophagy and mitochondria modulators in cancer treatment remains limited due to several challenges, including their lack of specificity, the development of resistance, cancer heterogeneity, and unclear molecular mechanisms underlying their actions. To address these issues, further studies are essential, especially those focused on discovering innovative drug targets and assessing synergistic combinations of drugs. Furthermore, creating autophagy-related biomarkers could help manage the variability among cancer patients and allow for more individualized treatment strategies.

## 6. Autophagy-Related Genes Hold Potential as Prognostic and Diagnostic Biomarkers for Cancer

Diagnosing and predicting the outcome of cancer in its early stages are essential for successful and effective treatment. Various types of cancer have unique autophagy-related biomarkers that serve as prognostic indicators. Identifying these biomarkers is essential for cancer diagnosis and can help predict the effectiveness of therapies that modulate autophagy.

In regard to melanoma, the extensively studied potential autophagy-related biomarkers are LC3, p62, and Beclin-1. Immunohistochemical analysis of malignant melanomas has revealed an increased expression of LC3 and decreased expression of Beclin-1, which are correlated with poorer patient outcomes and the progression of metastasis. However, there are instances of Beclin-1 overexpression and LC3 downregulation in advanced melanoma. The prognostic biomarker p62 is upregulated in the early stages of melanoma according to the AJCC (American Joint Committee on Cancer), but its expression is downregulated in advanced metastatic tumors [150]. In observational studies, these markers were validated in endometrial polyp tissue samples via immunohistochemistry (NCT04706000). Previous research on endometriosis has identified reduced levels of Beclin-1 mRNA and protein [151] (Table 4).

Analysis of mRNA expression in 52 normal and 495 tumor tissues from the Prostate Adenocarcinoma database identified mutations in *ATG9B*, *DNAJB1* (DnaJ heat shock protein family (Hsp40) member B1), *HSPB8*, *NKX2-3*, and *TP63* genes significantly associated with an increased risk of developing prostate cancer. Additionally, *BNIP3*, *NPC1*, and *TP53* genes serve as prognostic autophagy biomarkers for advanced stages of prostate cancer [152].

In oral squamous cell carcinoma (OSCC), RNA sequencing and clinical screening data analysis have identified *ATG12* and *BID* as potential prognostic autophagy-related biomarkers. Subsequent validation studies based on qRT-PCR, immunohistochemistry, and Western blot analysis have confirmed that these biomarkers are overexpressed in OSCC cell lines (SCC9, SCC15, SCC25) and tissues [153].

Research employing tissue microarrays, immunohistochemistry, and Western blot analysis conducted on formalin-fixed, paraffin-embedded tissues from 352 gastric cancer patients has indicated that diminished expression of ULK1, Beclin 1, ATG3, and ATG10 is associated with improved prognosis [154].

The transcriptome profiles from the TCGA (The Cancer Genome Atlas) and GTEx (The Genotype-Tissue Expression) databases, supported by clinical data and qPCR analysis of fresh cervical cancer samples, revealed *ATG4D*, *CD46*, *TP73*, and *HSPB8* as autophagy-related risk biomarkers. These markers are downregulated in cervical cancer and are associated with favorable prognosis [155]. An additional autophagy-related long non-coding RNA (lncRNA) involved in cervical cancer identified using a public database has established 10 lncRNAs with prognostic potential, with *DBH-AS1* being the most notable. Moreover, the associated study confirmed the role of lncRNA in regulating autophagy, modulating tumor development, and altering sensitivity to treatment [156].

In regard to glioma, one well-known biomarker is *VMP1*. Data analyses based on various cancer genome atlases have shown that *VMP1* is upregulated in high-grade gliomas, and this is associated with a worse prognosis. Suppressing *VMP1* expression through CRISPR-Cas9 gene editing significantly inhibited the proliferation of LN299 cells, leading to partial autophagy as a result of disrupted autophagosome formation and the initiation of apoptosis. *VMP1* has the potential to be utilized as a predictor of survival for glioma patients [157].

In regard to bladder cancer, 11 autophagy-related biomarkers have been identified as key indicators of patient survival and clinical outcomes based on information from the Human Autophagy Database and Bladder Carcinoma databases. These biomarkers are *APOL1*, *ATG4B*, *BAG1*, *CASP3*, *DRAM1*, *ITGA3*, *KLHL24*, *P4HB*, *PRKCD*, *ULK2*, and *WDR45*. Notably, the overexpression of *ULK2* and *P4HB* is linked to high-risk bladder cancer. In contrast, the overexpression of *APOL1*, *ATG4B*, *BAG1*, *DRAM1*, *ITGA3*, *KLHL24*, *PRKCD*, and *WDR45* is correlated with low-risk bladder cancer [158]. A study (NCT03254888) on bladder cancer patients with confirmed histopathology employed quantitative real-time PCR to estimate ATG7 levels and used immunohistochemistry to determine LC3A levels as markers of autophagy (Table 4).

In regard to esophageal cancer, RNA-sequencing data analysis and clinical information derived from TCGA database identified *DNAJB*, *BNIP1*, *VAMP7*, and *TBK1* (TANK binding kinase 1) as prognostic autophagy-related signatures. These biomarkers are significantly associated with overall patient survival [159].

Significant increases in autophagy and mitophagy markers, such as Beclin-1, LC-3, BNIP-3, and Parkin, were detected in breast cancer tissues compared to controls. The associated study also indicated that LC3 immunostaining was linked to younger breast cancer patients, while Parkin was associated with a history of breastfeeding [160].

An analysis of ovarian cancer gene expression profiles from the TCGA database, in conjunction with clinical data, uncovered 52 potential autophagy-related genes. LASSO-Cox analysis further revealed that FOXO1 and CASP8 are particularly promising for prognosis. Immunohistochemical analysis of tissue microarrays from 125 patients identified that elevated FOXO1 expression is linked to metastasis and a poorer prognosis in ovarian cancer [161].

Cancer prognosis and diagnosis are highly demanding disciplines owing to the diversity and intricacy of gene expression in individual patients. Each cancer patient possesses distinct genetic profiles and undergoes unique modifications in gene expression in response to cancer development. This uniqueness presents challenges in predicting cancer progression and treatment response. The heterogeneity in gene expression among cancer patients results in variation in tumor behavior and sensitivity to chemotherapy. Identifying specific prognostic biomarkers can contribute to early cancer patient diagnosis and enhance the effectiveness of personalized treatment strategies.

## 7. Conclusions

This review provides a comprehensive overview of the various functions of mitochondria within cancer cells, with a specific focus on their role in autophagy. Understanding the distinct characteristics of mitochondria in both healthy and cancerous cells, particularly in relation to autophagy, is crucial for developing more precise treatments, especially for cancer [9]. Strategies for inhibiting mitochondrial function or autophagy in cancer treatment include: 1. inducing oxidative stress; 2. disrupting mitochondrial respiration by targeting Complexes I through V; 3. inhibiting non-voltage calcium channels; 4. suppressing TCA cycle enzymes; and 5. modulating autophagy through activation or inhibition [162].

Autophagy is initiated under conditions of starvation and stress, such as organelle damage and the presence of misfolded proteins [163]. In cancer, autophagy plays a dual role: it can either promote tumorigenesis in certain cancers or suppress tumor development in others. Additionally, autophagy plays a role in the development of drug resistance and metastasis [12]. Modulating autophagy in cancer cells holds promise for cancer treatment. Autophagy inhibitors like chloroquine and hydroxychloroquine, as well as autophagy activators such as temsirolimus and rapamycin, have demonstrated efficacy in disrupting tumor growth, especially when combined with chemotherapy, according to both preclinical and clinical studies [164]. Despite the potential of mitochondrial function or autophagy modulators, their application is limited by several factors, including a lack of specificity, incomplete targeting due to mutations at binding sites, adverse events, and the development of secondary resistance.

Moreover, the precise diagnosis of specific cancer types is as crucial as the development of effective treatments. Early-stage cancer diagnosis significantly enhances the likelihood of successful treatment. Autophagy-related biomarkers are valuable for both cancer diagnosis and prognosis. This review also provides an overview of unique autophagy biomarkers across different types of cancer in pre-clinical and clinical studies.

## Figures and Tables

**Table 4:** Observational research on cancer patients derived from clinical trial database.

Title	Type of Study	Autophagy Markers/Evaluation	Type of Cancer	ClinicalTrials.gov ID
Investigation of autophagy markers in endometrial polyps (Turkey)	Observational	Beclin 1 LC3A/B P62/ Immunohistochemistry ELISA	Endometrial polyp	NCT04706000
Association of autophagy-related genes, LncRNA and SNPs with colorectal cancer in egyptian population (Egypt)	Observational	In PBMC and tissue, the levels of expression of EIF4EBP1, HOTTIP and serum SNP HOTTIP rs1859168	Colorectal cancer	NCT04729855
Identification of novel autophagy markers in bladder cancer patients (Egypt)	Observational	Atg7 (RTPCR) LC3A (immunohistochemistry)	Bladder cancer	NCT03254888
Immunohistochemical assessment of programmed death ligand 1 PDL-1 and autophagy marker LC3B in glioblastoma (Egypt)	Observational	LC3B (immunohistochemistry)	Glioblastoma	NCT04284306

## Abbreviations

13OD	13-oxyingenol-dodecanoate
ACD	autophagic cell death
AJCC	American Joint Committee on Cancer
AKT1	AKT serine/threonine kinase 1
AMBRA1	the activating molecule in BECLIN1-regulated autophagy 1
AMPK	AMP-activated protein kinase
ATP	adenosine triphosphate
ATP5F1A	ATP synthase F1 subunit alpha
BAG1	Bcl2-associated athanogene 1 protein
BCL-2	B-cell lymphoma-2
BCL2L13	BCL2-Like 13
Beclin1	Bcl-2-interacting myosin-like coiled-coil
BIF1	Bax-interacting factor 1
BMAL1	basic helix–loop–helix ARNT-like 1
BNIP3	BCL2/adenovirus E1B 19 kDa protein-interacting protein 3
CALCOCO2/NDP52	calcium-binding and coiled-coil domain 2
CAT	catalase
CHIP	carboxyl terminus of hsc70-interacting protein
CHMP2	charged multivesicular body protein 2
CL	cardiolipin
CLEAR	coordinated lysosomal expression and regulation
CMA	chaperone-mediated autophagy
COX IV	cytochrome c oxidase subunit 4
DAPK	death-associated protein kinase
DAPK	death-associated protein kinase
DJ-1	nucleic acid deglycase
DNAJB1	DnaJ heat shock protein family (Hsp40) member B1
DRAM	damage-regulated autophagy modulator
DUB	deubiquitinase
DYNC1LI2	Dynein Cytoplasmic 1 Light Intermediate Chain 2
EF1α	elongation factor 1-α
EGCG	epigallocatechin gallate
ER	endoplasmic reticulum
ESCRT	endosomal sorting complex required for transport
ETC	electron transport chain
FKBP8	FKBP Prolyl Isomerase 8
FUNDC1	FUN14 Domain-Containing 1
GABARAP	GABA type A receptor-associated protein
GFAP	glial fibrillary acidic protein
GP78	glycoprotein 78
GSH	glutathione
GSH-Px	glutathione peroxidase
GTEx	genotype-tissue expression
GTP	guanosine triphosphate
HMT	horizontal mitochondrial transfer
HOP	hsp70-hsp90 organizing protein
HOPS	homotypic fusion and protein sorting
Hsp40	heat shock protein 40
Hsp70	heat shock protein 70
IMM	inner mitochondrial membrane
KGDH	α-ketoglutarate dehydrogenase
LAMP-2A	lysosome-associated membrane protein type 2A
LC3	microtubule-associated protein 1 light chain 3
LIR	LC3-interacting region
MAM	mitochondria-associated membrane
MAP1LC3/LC3	microtubule associated protein 1 light chain 3
MFN2	Mitofusin-2
Miro	mitochondrial Rho GTPase
MMP	mitochondrial membrane potential
MTCH1	mitochondrial carrier 1
mTORC1	mechanistic target of rapamycin complex 1
mTORC2	mammalian target of rapamycin complex 2
mtROS	mitochondrial reactive oxygen species
MUL1	mitochondrial E3 ubiquitin ligase 1
NBR1	neighbor of BRCA1 gene 1 protein
NFAT1	nuclear factor of activated T cells
NFE2L2	NFE2 Like BZIP transcription factor 2
NF-kB	nuclear factor kappa-light-chain-enhancer of activated B cells
NOX	NADPH oxidase
Nrf2	nuclear factor erythroid 2-related factor 2
OMM	outer mitochondrial membrane
OPTN	optineurin
OSCC	oral squamous cell carcinoma
OXPHOS	oxidative phosphorylation
PAS	pre-autophagosomal structure
PDH	pyruvate dehydrogenase
PE	phosphatidylethanolamine
PER1/2	period circadian protein homolog 1/2
PGAM5	mitochondrial serine/threonine protein phosphatase
PHB2	prohibitin 2
PHLPP1	PH Domain And Leucine Rich Repeat Protein Phosphatase 1
PI3K	phosphatidylinositol 3-kinase
PI3P	phosphoinositide 3-phosphate
PINK1	PTEN-induced putative kinase 1
PLEKHM1	pleckstrin homology domain-containing family M member 1
PPT1	palmitoyl-protein thioesterase 1
PTEN	phosphatase and tensin homolog
Rab-7A	Ras-related protein Rab-7A
RARα	retinoic acid receptor alpha
RHOT1	Ras homolog family member T1
RILP	Rab-interacting lysosomal protein
ROS	reactive oxygen species
SIAH1	seven in absentia homolog 1
SMAC	mitochondrial-derived activator of caspases
SNARE	soluble N-ethylmaleimide-sensitive attachment protein receptors
SOD	superoxide dismutase
SQSTM1/p62	sequestosome 1
TAX1BP1	Tax1 binding protein 1
TBK1	TANK binding kinase 1
TCA	tricarboxylic acid
TCGA	The Cancer Genome Atlas
TFEB	transcription factor EB
TLR	toll-like receptor
TNFa	tumor necrosis factor α
Tom20	mitochondrial import receptor subunit TOM20 homolog
TOMM7	translocase of outer mitochondrial membrane 7
TRAF6	tumor-necrosis-factor-receptor-associated factor 6
TRX	reduced thioredoxin
TRX-Px	thioredoxin peroxidase
TSC1/2	tuberous sclerosis protein 1 and 2
ULK	Unc-51-like kinase
ULK1/2	serine/threonine Unc-51 like kinase ½
UVRAG	UV radiation resistance-associated
VDAC-1	voltage-dependent anion channel-1
VPS34	vacuolar protein sorting 34
WIPI2	WD-repeat domain phosphoinositide-interacting 2
